# Advanced 3D Imaging of Uterine Leiomyoma’s Morphology by Propagation-based Phase-Contrast Microtomography

**DOI:** 10.1038/s41598-019-47048-0

**Published:** 2019-07-22

**Authors:** Alessandra Giuliani, Stefania Greco, Serena Pacilè, Alessandro Zannotti, Giovanni Delli Carpini, Giuliana Tromba, Stefano Raffaele Giannubilo, Andrea Ciavattini, Pasquapina Ciarmela

**Affiliations:** 1 Department of Clinical Sciences, Università Politecnica delle Marche, Ancona, Italy; 2 Department of Experimental and Clinical Medicine, Università Politecnica delle Marche, Ancona, Italy; 3 Elettra Sincrotrone Trieste S.C.p.A, Trieste, Italy

**Keywords:** Biophotonics, Infertility

## Abstract

Uterine leiomyoma is the most common benign smooth muscle tumor in women pelvis, originating from the myometrium. It is caused by a disorder of fibrosis, with a large production and disruption of extracellular matrix (ECM). Medical treatments are still very limited and no preventative therapies have been developed. We supposed that synchrotron-based phase-contrast microtomography (PhC-microCT) may be an appropriate tool to assess the 3D morphology of uterine leiomyoma, without the use of any contrast agent. We used this technique to perform the imaging and the quantitative morphometric analysis of healthy myometrium and pathologic leiomyomas. The quantitative morphometric analysis of collagen bundles was coupled to the Roschger approach. This method, previously only used to evaluate mineralized bone density distribution, was applied here to study the fibrosis mass density distribution in healthy and pathologic biopsies from two patients. This protocol was shown to be powerful in studying uterine leiomyomas, detecting also small signs of the ECM alteration. This is of paramount importance not only for the follow-up of the present study, i.e. the investigation of different compounds and their possible therapeutic benefits, but also because it offers new methodologic possibilities for future studies of the ECM in soft tissues of different body districts.

## Introduction

Uterine leiomyoma (myoma or fibroid) is the most common benign smooth muscle tumor in women pelvis causing significant morbidity in a large segment of reproductive-aged women^1,2^, including significant impact on the reproductive healthy status and on the pregnancy outcome of affected patients^3,4^.

Fibroids remain the leading indication for hysterectomy^5,6^ especially during the peri-menopause period, although the assumption that leiomyoma symptoms will resolve with the onset of the menopause is too simplistic and may not be always valid^7^. Medical treatments for leiomyoma are still very limited and no preventative therapies have been developed^8^.

The precise pathogenesis of uterine leiomyoma is not well understood. Genetic alterations, epigenetic mechanisms, steroids, growth factors, cytokines and chemokines provide the clue of initiators and promoters of leiomyoma growth^9^. Uterine myoma is thought to be a consequence of myofibroblasts activation and extracellular matrix (ECM) production, following an improper inflammatory response inside the myometrium^10–12^. Excessive accumulation of ECM components including collagens, fibronectin and proteoglycans are the major structural part of leiomyoma tissue that are abnormally orientated modifying mechanical stress on cells and leading to activation of internal mechanical signaling and dynamic reciprocity^13–15^. The ECM stiffness causes the rigid structure of the leiomyoma and it is thought to be a cause of abnormal bleeding and pelvic pressure and pain^16^.

The role of inflammation and reparative processes as well as the effect of some potential therapeutic compounds (tranilast, strawberry extracts, omega-3 fatty acids, genistein, natural fitoterapeutics, vitamin D) on uterine leiomyoma are currently under *in vitro* investigation^17–29^.

At the moment, structural laboratory studies of myometrial and fibrotic tissue are limited to the two-dimensional (2D) morphological investigation. Only an advanced three-dimensional (3D) characterization of the fibrotic tissue structure can enhance our knowledge of the tissue architecture, significantly supporting the development of an effective therapeutic solution. For example, the *in-vivo* efficiency of therapeutic compounds could be hampered, even if delivered locally, by their inability to reach the leiomyoma cells due to the fibrotic hard and perhaps not permeable tissue architecture. Therefore, it would be essential to evaluate the whole 3D tissue structure.

Nowadays, X-ray tomography (CT) is not the method of choice for the characterization of pelvic masses; indeed, uterine fibroids are detected incidentally by CT scans, often performed for other reasons. Medical ultrasonography (USG) is usually the initial investigation tool to examine the female pelvis, performing both transabdominal and transvaginal scans. Magnetic resonance imaging (MRI) is the favourite method for an accurate characterization of pelvic masses. Indeed, MRI was shown to be more sensitive in identifying uterine fibroids than USG, and it has the advantage, respect to CT, that it does not involve the use of ionizing radiation^30,31^.

However, up to now, literature has not definitely clarified if the discrimination between healthy and pathologic myometrium is just a matter of stiffness or there are also any density variations. For instance, the typical CT images show fibroids that may appear morphologically complex, with hypodense, though they may be isointense, and rarely hyper intense areas. Calcification is seen in approximately 4% of fibroids: it is typically dense and amorphous, sometimes confined to the periphery of the fibroids. As such, the CT appearance of leiomyomas cannot reliably be distinguished from uterine or cervical neoplasms^32^.

X-ray phase-contrast imaging (XRPCI) techniques may constitute a potential powerful tool for non-invasive myometrium imaging, in healthy and pathologic conditions and without the use of any contrast agent. Indeed, differently from the X-ray absorption-based imaging, where the contrast originates from attenuation mismatches between different tissues inside a sample, in the XRPCI the contrast is due to the phase-shift δ of the refractive index n = 1 − δ + iβ, describing the interaction of the X-ray beam throughout the material^33,34^. This δ value, in non-mineralized biological tissues like the myometrium, can be up to three orders of magnitude larger than attenuation values β^35,36^, achieving an highly increased contrast, as observed investigating several organs/tissues, including brain^37,38^, vessels^39–41^, kidney^42^, neuronal system^43^, cartilage^44,45^ and breast tissues^46,47^. Moreover, ten years ago, synchrotron radiation-based high resolution phase-contrast tomography (SR-PhC-microCT) was also used, in a pioneering way, for the study of the ECM organization within polymeric scaffolds^48^.

No XRPCI studies, to the proponents’ best knowledge, have been performed so far on the uterine myometrium and leiomyomas without the use of a contrast agent. Thus, in the present demonstrative study, we aimed to test the use of the propagation-based phase-contrast imaging to discriminate healthy and pathologic uterine tissues.

## Results

Taking into account that the slight nominal mismatch between collagen (1.41 g/cm^3^)^49^ and muscle (1.055 ÷ 1.112 g/cm^3^)^50^ mass densities would have prevented, most likely, a reliable analysis by absorption-based imaging, all the 15 biopsies were investigated by PhC-microCT and subsequently reconstructed exploiting the Paganin’s method^51^ for the phase retrieval processing.

**Figure 1:**
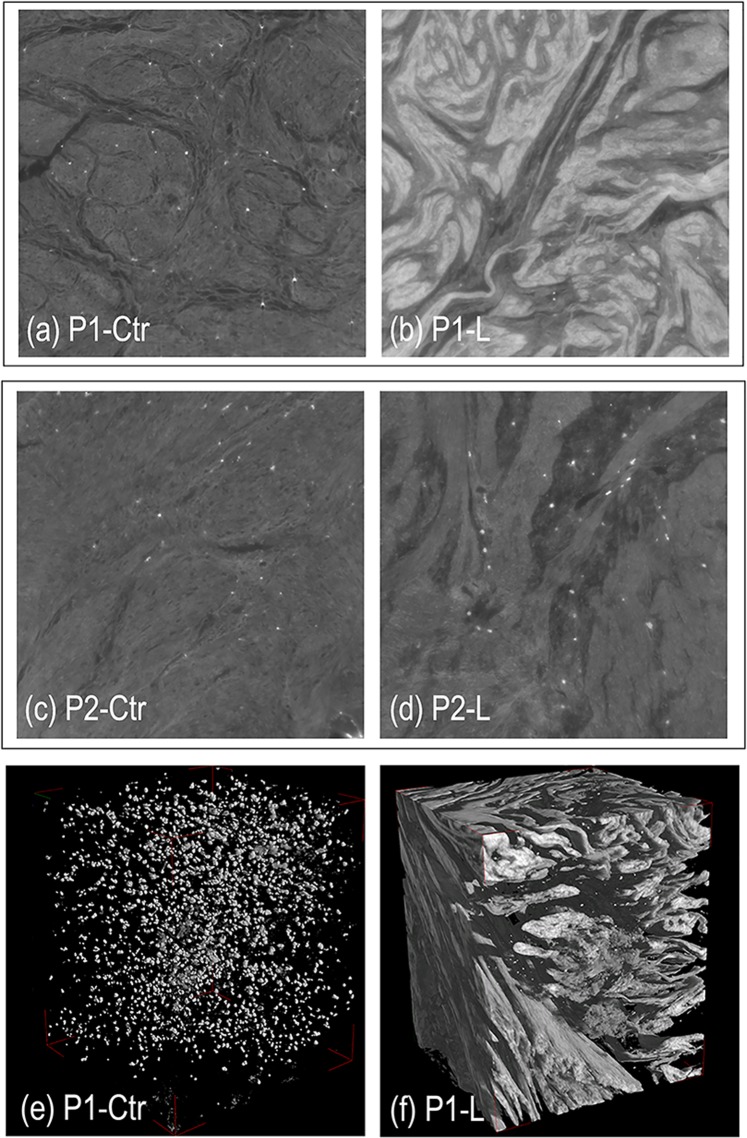
(**a**–**d**) Representative 2D slices of healthy myometrium (Ctr) and pathologic (L) tissues. (**a**,**b**) Patient 1 (P1): healthy myometrium (**a**: P1-Ctr) and leiomyoma (**b**: P1-L) tissues; (**c**,**d**) Patient 2 (P2): healthy myometrium (**c**: P2-Ctr) and leiomyoma (**d**: P2-L) tissues. Morphological dissimilarities were found not only between Ctr and L samples of the same patient and between pathologic tissues of the two patients (P1-L vs. P2-L) but also between the healthy tissues of the two patients (P1-Ctr vs. P2-Ctr). (**e**,**f**) Representative 3D volumes of healthy myometrium (**e**: P1-Ctr) and pathologic (**f**: P1-L) tissues: all the tissues but the collagen phase have been made virtually transparent. The bright spots that we found in all the samples were due, most likely, to the presence of blood clots residues, not removed by washing because of the structure tortuosity.

Representative 2D slices of healthy myometrium (Ctr) and leiomyoma (L) tissues are shown in Fig. 1 (panels a–d) for both patients P1 and P2. Stack-sequences of 2D axial slices (subvolumes, each with a final volume of 600 × 600 × 400 µm^3^) have been reported for representative samples of P1 in Movies 1 and 2 in the Supplementary Material section. 3D reconstructions of the same volumes are shown in Fig. 1 (panels e,f), where all the tissues but the collagen phase have been made virtually transparent. Morphological dissimilarities were clearly found between Ctr and L samples of the same patient (P-Ctr vs. P-L) and between pathologic tissues of the two patients (P1-L vs. P2-L). The analysis was also able to reveal differences between the healthy tissues of the two patients (P1-Ctr vs. P2-Ctr).

In agreement with previous literature, mainly based on histologic findings, the pathologic biopsies revealed an higher amount of collagen than the healthy myometrium. Moreover, distribution and orientation of the collagen bundles were very varied in the different samples, suggesting the need for a detailed morphometric analysis.

The quantitative analysis was performed with an approach based on two levels of investigation.

**Figure 2:**
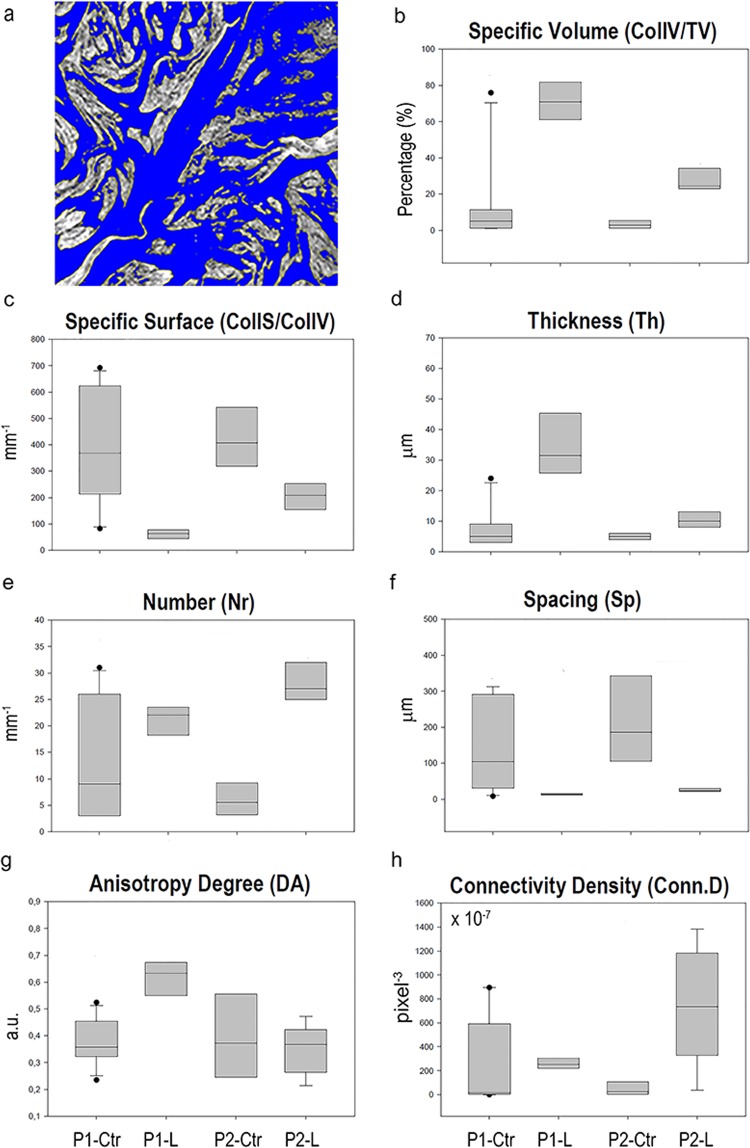
1st level analysis: study of the collagen morphometric quantification. (**a**) Sampling 2D slice of a pathologic biopsy where the histogram has been segmented according to the application of the Mixture Modeling algorithm. Blue phase: smooth muscle; graded grey phase: collagen bundles. (**b**–**h**) Box-plots graphically depicting groups of the extracted collagen morphometric parameters through their quartiles, as a function of the selected groups of samples P1-Ctr, P1-L, P2-Ctr and P2-L.

In the first level, the application of the Mixture Modeling algorithm^52^ to the histograms (based on an unsigned 8-bit scale of grey) of the full set of samples indicated a threshold of 110 between muscle and collagen grey levels, achieving the collagen morphometric quantification, as detailed in Table 1 and illustrated in Fig. 2.

**Table 1:** Collagen three-dimensional morphometric analysis in the retrieved biopsies.

	P1-Ctr	P1-L	P2-Ctr	P2-L
CollS/CollV [mm^−1^]	388 ± 213	63 ± 20	422 ± 117	200 ± 71
CollV/TV [%]	15.0 ± 24.4	70.5 ± 10.9	3.1 ± 2.1	32.6 ± 15.8
Th [μm]	7.8 ± 6.8	35.4 ± 13.1	5.0 ± 1.2	11.4 ± 4.9
Nr [mm^−1^]	13.0 ± 10.7	21.1 ± 3.8	6.0 ± 3.2	28.7 ± 4.4
Sp [µm]	145.9 ± 125.2	13.9 ± 3.7	211.3 ± 126.4	23.9 ± 7.1
DA	0.378 ± 0.081	0.632 ± 0.089	0.399 ± 0.166	0.378 ± 0.081
Conn.D [×10^−7^ pixel^−3^]	234.6 ± 368.5	259.4 ± 52.5	45.0 ± 58.3	741.9 ± 472.2

Mean values ± standard deviation. P1-Ctr: heathy myometrium in patient 1; P1-L: leiomyoma in patient 1; P2-Ctr: healthy myometrium in patient 2; P2-L: leiomyoma in patient 2.

The specific surface (CollS/CollV) and the mean distance between the collagen bundles (Sp) in the leiomyoma were lower than in the healthy myometrium. Coherently, the specific volume (CollV/TV), the mean thickness (Th) and the mean number (Nr) of collagen bundles were higher than in the healthy myometrium.

Unexpectedly, three other results were obtained, as detailed in Table 1:

1. although the number of samples analyzed was comparable, the standard deviation of data obtained for P1-Ctr was always higher than for data related to P2-Ctr;
2. the Nr was higher in P1-Ctr than in P2-Ctr;
3. the Connectivity Density (Conn.D) was comparable between P1-Ctr and P1-L but lower in P2-Ctr than in P2-L.

In general, these three results, revealed large structural inhomogeneity inside myometrial and leiomyoma tissues.

Moreover, up to now, literature has not definitely clarified if the discrimination between healthy and pathologic myometrium is just a matter of stiffness or there are also density variations. Consequently, we achieved the second level of the quantitative analysis. It was based on the approach designed by Roschger^53^ and, to date, it was succesfully used to evaluate the mineral density distribution in bone (BMDD)^54,55^. Indeed, the Roschger method delivered fundamental descriptors of the mineral density distribution of the bone matrix throughout a sample: thus, it was shown to play a fundamental role in testing if either diseases and/or treatments might be of significant biological and clinical relevance^53^.

**Figure 3:**
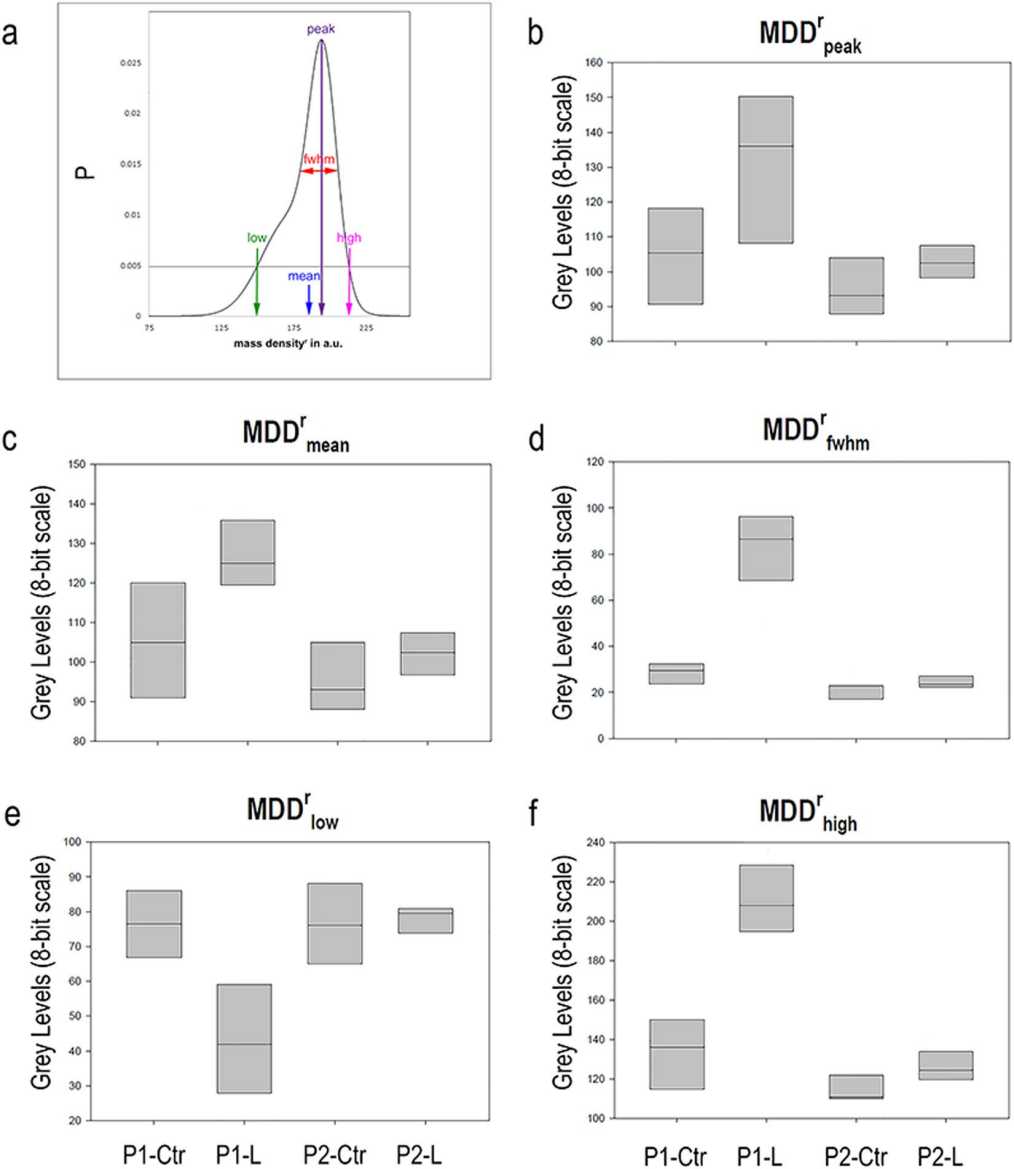
2nd level analysis: study of the Relative Mass Density Distribution (MDD^r^). (**a**) The parameters derived from the profile fitting are indicated. (**b**–**f**) Box-plots graphically depicting the extracted MDD^r^ parameters through their quartiles as a function of the selected groups of samples P1-Ctr, P1-L, P2-Ctr and P2-L.

Thus, starting from the rational that the leiomyoma pathology was shown to be connected to the excessive accumulation of collagen, the study of fibrosis mass density distribution by the Roschger’s approach was supposed to help in clarifying if distribution deviations from healthy conditions were involved. We tested the Roschger method descriptors (Fig. 3a) to evaluate, for the first time to the authors’ knowledge, the apparent uterine mass density distribution (MDD^r^). Similarly to the previous studies, absolute values of mass density could not be retrieved because they might have been biased by the used constant delta-over-beta ratio in the Paganin phase retrieval^56^. However, being the different biopsies comparable in terms of size and composition, the quantitative comparison of the relative difference in mass density distribution between the samples is feasible. Thus, hereinafter the superscript r will denote relative values for all mass density distribution parameters.

The quantitative data obtained in the investigated biopsies were reported in the box plots of the Fig. 3b–f. Median values of the relative mass density distribution in P1-Ctr were found lower than in P1-L; on the contrary, the fwhm and the high (99.5th percentile) values were higher in the leiomyoma in comparison to the healthy controls for both the patients. However, as expected after the morphometric analysis of collagen, the fwhm and the high values of P1-Ctr were higher than those obtained in P2-Ctr. Therefore, it must be concluded that some or all the biopsies derived from patient 1, clinically considered non-pathological tissue portions, were actually composed by very heterogenic samples, in terms of stiffness and density distribution.

## Discussion

The role of the extracellular matrix (ECM) in the biomechanics of the human uterus has just been begun to be studied in recent years. The ECM does not only surround cells, but its rigidity stresses them mechanically, producing signals that depend on the amount of collagen, its cross-linking and hydration. Therefore, the ECM can induce the so-called mechano-transduction process, namely cells convert a mechanical stimulus into an electrochemical activity. Indeed, the etiology of uterine fibroids indicates that its growth is due to an increase not only of the cell number but also of the ECM amount, both promoted by endocrine and autocrine growth factors. In turns, the alterations in ECM volume and distribution can modify mechanical stress on cells, leading to activation of specific cell’s signaling that contribute to leiomyoma growth^16,57^. Therefore, alterations of morphology and/or composition of the ECM can be important signals of the onset and of the evolution of this pathology.

Previous studies showed an impaired expression of collagen, fibronectin and proteoglycans in leiomyoma compared to normal myometrium^58–61^: in particular, leiomyoma was shown to contain an abnormal collagen fibril structure and orientation, suggesting that the well-regulated fibril formation in myometrium is altered in leiomyomas. In fact, an important study showed that the interaction of two or more different types of fibrillar collagen chains may interact and result in the formation of heterotypic fibrils, missing in leiomyomas, that in myometrium would assist in regular fibril formation in normal uterine tissue. In this context, alterations in collagen may play a role in the pathogenesis of leiomyomas^62^. Thus, the study of specific collagen parameters, such as the size of collagen bundles, orientation and interconnectivity, turns out to be particularly interesting because its morphometry should determine specific cellular behaviors. This is similar to what happens in bone sites, although the interpretation of what morphometric measurements mean for collagen masses in the myometrium must be related to the specific myometrial tissue and its function.

Therefore, we have focused our analysis on the study of collagen. The propagation-based phase-contrast microCT was used, for the first time to the authors’ knowledge, to evaluate the collagen amount and distribution in intramural fibroids excised from the uterus of two fertile women.

In our study, we observed higher volumes, thickness and number of collagen bundles in the leiomyoma compared to normal adjacent myometrium. In the case of patient 1, the interconnectivity of the collagen structure was also increased in the pathological tissue compared to the control. These data obtained on collagen networks justify the increase in the rigidity of the pathological tissue compared to the healthy one and they are in agreement with the observation performed with conventional 2D techniques, like histology and electron microscopy, in several studies found in literature.

The specific surface (CollS/CollV), defined as the surface area per unit volume, is an architectural parameter that can contribute to determine type and properties of a material. This measure is strongly dependent from shape: if volume is held constant, matrix of different shapes will typically adopt different specific surface values. Interestingly, we found that collagen specific surface in leiomyoma is lower than in normal counterpart. Certainly, this is due to the fragmentation of collagen in myometrium versus the aggregation of collagen in leiomyoma. To the authors’ knowledge, collagen specific surface has never been measured in myometrium or leiomyoma tissue, although it could represent an important index to study, due to relevant clinical implications. The presence of focal nodal collagen distribution in the myometrium, as in the case of uterine fibroids, could alter the normal uterine peristalsis through mechanical interference. Indeed, it was reported that patients with intramural fibroids may present abnormal uterine peristalsis during the mid-luteal phase. Since uterine peristalsis is involved in normal reproductive processes, this condition seems to reduce the possibility of obtaining spontaneous pregnancy^63^. Moreover, the study from Yoshino and colleagues highlighted how myomectomy reduced the frequency of abnormal peristalsis in all patients^64^. Collagen specific surface may become a useful parameter to perform further studies, i.e. comparing normal myometrial tissues versus fibroid tissues, as well as to study different type of fibroids (i.e. large vs small or pre-menopausal vs post-menopausal or benign vs malignant).

Although the present study should be considered prevalently as a set-up of a new study methodology, we consider appropriate to point out that the structural properties of fibroids and even of healthy myometrium tissues differ widely within the same tissue and among the different patients.

We and others have recently observed that the amount of collagen inside tissues within and between biopsies is heterogeneous mainly in leiomyoma, but also in myometrial tissue. For example, morphometric analysis of Masson’s trichrome stained tissue reported values of collagen in leiomyoma ranging from 4.7% to 31,3% in our previous study^11^; from 37% to 77% in Jayes^65^ study and from 3.9% up to 70% in a large study of brazilian cases (n = 150) performed by Da Silva^66^. Flake and colleagues categorized uterine leiomyoma in 4 different phases on the basis of percent collagen content ranging from none (phase 1) up to 72% (>50%, phase 4) independently of the tumor size^67^. The collagen content in myometrium ranged from 0.3% to 23% in our previous samples^11^ and from 1.6% up to 20.70% in Da Silva’s study^66^. Interestingly, the leiomyoma tissues appeared inhomogeneous also in stiffness, quantified through rheometry by measuring complex shear moduli of the tissues. Fibroid tissues, with stiffness of 8014 ± 798 Pa (mean ± SEM), were stiffer than myometrial samples (3630 ± 276 Pa)^65^. With the same indication, evidence of biomechanical and biochemical heterogeneity in uterine fibroids has just been reported. The authors observed heterogeneity in structure, collagen content, and stiffness highlighting that fibroid regions differ in biochemical status and they suggest that these differences might be associated with variations in local pressure, biomechanical signaling, and altered growth^68^.

All this opens new prospective to understand uterine fibroids and to explain how it is possible that, despite of the same hormonal milieu, different fibroids of the same subject have a different fate and degree of growth. These differences in fibroids structure could also explain the heterogeneous effect of fibroids of the same dimensions, number, and localization on fertility, on response to medical therapy, and on pregnancy outcomes.

Therefore, the present and all these recent studies, provide novel evidences that structural properties of uterine leiomyoma and, even if less pronounced, also of the myometrium are widely heterogeneous. The variability within and among tissues should be considered in order to characterize the samples, to design and conduct studies to understand the pathobiology as well as to test potential treatments.

Moreover, it has to be stressed that physical, topological, and biochemical composition of the ECM is not only tissue-specific, but it is also markedly heterogeneous: ECM is a highly dynamic structure that is constantly being remodeled, as it happens in bone sites. For instance, collagen density distributions in human knee ligaments were documented to quantify differences in density within and between these ligaments^69^. Very recently, heterogeneity in structure, collagen content, and stiffness in uterine fibroids was reported^68^. Thus, it is interesting to evaluate if this heterogeneity is expressed also in terms of density distributions, as it was shown in human knee ligaments.

In our study, we performed the analysis of fibroid mass density distribution by the Roschger’s approach. We found that the full width at half maximum and the 99.5th percentile values were significantly higher in the leiomyoma in comparison to the healthy adjacent controls for both the patients. This means that, in pathological biopsies, not only the amount of collagen is greater (as found out by the morphometric analysis) but also its density distribution is wider than in controls, with presence of highly fibrotic and high-density areas.

Another remark that deserves to be made concerns the density variability of healthy myometrial tissue.

Indeed, using the Roschger approach we showed that, comparing healthy myometrial tissues, the full width at half maximum and the 99.5th percentile values of patient 1 were higher than the respective ones obtained in patient 2, confirming that some tissue portions clinically classified as healthy, showed not only differences in ECM volume but also in density distribution.

On the other hand, it should not be surprising to realize that myometrium of fibromatous uterus is not a homogeneous tissue; it presents structural and functional alterations and consequently it cannot be considered completed healthy. For example, just consider that in the myometrium surrounding the leiomyoma there is accumulation of inflammatory cells^11^. Furthermore, irregular thickening of the endomyometrial junctional zone due to inordinate proliferation of the inner myometrium, junctional zone hyperplasia, is a common MR finding in women suffering from menstrual dysfunction^70,71^.

In this context, a textural analysis would have been additionally informative; however, its application in this demonstrative study would have been challenging and perhaps not completely reliable. Indeed, in order to have a reliable textural analysis, the pixel size should be decreased to at least 500–600 nm, in order to fully resolve the texture also in the healthy myometrium (in Fig. 1e the spotty-signal reveals only the larger nodes of the collagen structure).

In conclusion, the propagation-based phase-contrast microCT was shown to be a powerful method in studying uterine leiomyomas, detecting also small signs of the ECM alteration.

In general, the PhC-microCT was shown to provide 3D images of intact tissues as well as a wide range of numerical indices that can be calculated and used to identify mismatches between different tissues. This is of fundamental importance with a view to follow up these studies in various directions: first of all, to visualize the 3D structure of the ECM in myometrium and pathologic tissues (different forms of leiomyoma, as well as leiomyosarcoma); secondly, to test *ex-vivo* in toto tissue the efficacy of innovative therapeutic treatments for leiomyomas; more generally, to offer new methodological possibilities for future studies on ECM in soft tissues of different body districts.

## Methods

### Sample collection and Permissions

Samples of myometrial tissue and leiomyoma were excised from two fertile women submitted to hysterectomy. The patients were Caucasians (age: 49 years and 43) and the position of the leiomyomas was intramural: the former with a single mass of 10 cm, the latter with two masses of 4 cm and 2 cm. Both patients displayed good general condition; none of them had a history of myomectomy or uterine surgery, had received medical therapy or oral contraceptives in the previous three months, or had evidence of genital tract infection, endometriosis, or ovarian disease. Patients gave their informed consent and the permission of the Human Investigation Committee was granted (Ethics Committee of Marche Region, Prot. N 2015 0486OR).

All experiments were performed in accordance with relevant guidelines and regulations.

The samples, immediately after hysterectomy, were collected in Hanks’ Balanced Salt Solution (HBSS) (Euroclone, Milan, Italy) and transferred to the laboratory for washing them with Dulbecco’s PBS (Invitrogen, Life Technologies, Carlsbad, CA, USA) in order to remove excess of blood and cutting them into small pieces.

In the former patient (P1), four pieces were taken from the fibrotic tissue (P1-L) and other four from the healthy myometrium (P1-Ctr); in the latter patient (P2), four pieces were taken from the leiomyoma (P2-L) and other three from the healthy myometrium (P2-Ctr).

### Synchrotron Radiation microCT examination

The SR-microCT experiments were performed at the SYRMEP beamline of the ELETTRA Synchrotron Facility (Basovizza, TS, Italy).

SR-microCT in its simplest form is based on the inversion of the Radon transform of the experimental projections. After the so-called flat fielding of the projection, the filtered back projection (FBP) algorithm is applied to the corrected data in order to reconstruct the stack of 2D slices^72^. The conventional x-ray imaging approach is based on the discrimination of the different attenuation properties of the elements composing the imaged object, that are related to β, the complex part of the index of refraction n = 1 − δ + iβ. In this case, image contrast is generated by differences in x-ray absorption. However, because of the coherence of SR, also the electron density, related to the phase shift term δ, might be exploited leading to phase-contrast imaging^33^. With this approach, applicable only if X-ray wavefield has high coherence characteristics, the setup is sensitive to detect not only the absorption differences but also the phase shifts occurring to x-rays crossing the sample. This approach becomes fundamental when the discrimination between two materials having similar electron densities or negligible X-ray absorption is required. Indeed, the reconstruction of δ distribution improves the image contrast, thus simplifies the image segmentation and the subsequent quantitative analysis.

We used this last method to investigate the 3D morphology of the fibrotic tissue and the mass density distribution in the biopsies retrieved from patients affected by leiomyoma.

The scans were performed using the pink SR beam provided by the machine, filtered by 0.5 mm Silicon and corresponding to an average energy of 19 keV; we used 0.2 s of exposure time per projection over a total range of 180°; the sample-detector distance was set to 100 mm, resulting in 1 µm^3^ isotropic voxel size in the reconstructed 3D images. The tomographic reconstruction was performed using the SYRMEP Tomo Project (STP) open source software^72^. Including the intensity of the recorded radiographs a phase contrast signal, a phase retrieval algorithm, exploiting the Paganin’s method^51^, was applied to the (flat-corrected) projection data in order to reconstruct the decrement δ of the refractive index n. In the Paganin’s method, the phase is retrieved by simply assuming a linear relationship between the absorption index β and the refractive index decrement δ. The approximation is valid for homogenous samples and propagation distances in the near field regime^51^, conditions fulfilled for the considered samples and the adopted scans geometries. The δ/β ratio was set to 100.

Afterwards, the commercial software VG Studio MAX 1.2 (Volume Graphics, Heidelberg, Germany) was used to generate 3D images, where grey levels were proportional to the phase distribution. Optimal image quality was achieved by setting the Scatter HQ algorithm with an oversampling factor of 5.0.

### Image analysis

The presence of different phases within the biopsies translated into different peaks in the gray-level scale. Thus, the volume of each phase was obtained by multiplying the volume of a voxel by the number of voxels underlying the peak associated with the relevant phase. The Mixture Modeling algorithm, implemented as plugin in the NIH ImageJ software (https://imagej.net/Mixture_Modeling_Thresholding and https://imagej.nih.gov/ij/plugins/mixture-modeling.html)^73^, was chosen to threshold the portion of the histograms related to the two adjacent main tissues, namely the smooth muscle and the fibrotic collagen-based phase. The Mixture Modeling algorithm is a histogram-based technique that assumes that the histogram distribution is represented by two Gaussian curves. It calculates the image threshold as the intersection of these two Gaussians, finding a threshold that is, in several cases, very close to real world data. In our histogram data, the Gaussian curve on the left was representative of the smooth muscle, the second curve on the right of the collagen-phase. Indeed, in this range of densities should be included also the endothelial signal but the thickness of vessels, if excluding the pregnancy period, is negligible, not affecting the segmentation process. Therefore, we analyzed in each pathological biopsy, with the aforementioned algorithm, a dozen slices, uniformly selected in the axial direction: the selected threshold value was the average of all those obtained. Notably, for the selection of this threshold value, control biopsies were not examined because they were considered unreliable due to their low collagen content. An example of histogram segmented by the Mixture Modeling algorithm has been reported for a representative sample of P1-L in Fig. 4 of the Supplementary Material section.

For each biopsy, several subvolumes were analyzed: each of them was a 3D portion fully included in the sample bulk and the complete set of them allowed to achieve the complete sample mapping.

The quantitative analysis was performed in two steps.

In the first step, the morphometric evaluation of the fibrotic collagen-based phase was made using the structural indices usually measured for bone samples^74^: the collagen specific volume (CollV/TV, expressed as a percentage), its specific surface (CollS/CollV, per millimeter), the mean collagen bundles thickness (Th, expressed in micrometers), the mean collagen bundles number (Nr, per millimeter) and the mean collagen bundles spacing (Sp, expressed in micrometers). Furthermore, as collagen bundles could vary their orientation depending on the pathology, we also extracted information about the anisotropy of the collagen structure, i.e. the presence of preferential orientation(s). The anisotropy degree index (DA) measures the similarity of a fabric to a uniform distribution and varies between 0, representing all observations confined to a single plane or axis, and 1, corresponding to the perfect isotropy. The DA analysis was performed using the BoneJ Plugin^75^ of the ImageJ software^73^, version 3. The morphometric analysis was also extended to the inclusion of a descriptor for the interconnectivity of the collagen bundles: its connectivity density (Conn.D – pixel^−3^) does not carry information about positions or size of connections, but it is a simple global measure of connectivity that gives higher values for better-connected structures, i.e. more entangled fibrotic tissues, and lower values for poorly connected ones, i.e. well-oriented bundles.

In the second step, the reconstructed complex refractive index distribution was exploited^54^; indeed, it is linearly related to the mass density and was assessed computing the relative mass density distribution (MDD^r^) of each sample, as shown in Fig. 3a. In each slice-image, the individual grey level voxels reflect the average density found in the corresponding volume elements of the investigated sample region: consequently, the acquisition of grey level voxel images provided the information on the local variation of density throughout the tissue. This variation in density is best described and quantified by a frequency distribution (histogram), as shown in the Fig. 3a. In order to compare different mass density distributions between biopsies, a reduction of the huge amount of histogram data to only few characteristic parameters is necessary. Briefly, the MDD^r^ was calculated within the overall tissue domain and was normalized by the area under the curve.

The absolute values of mass density could not be retrieved because the reconstructed complex refractive index might be biased by the chosen δ/β ratio used in the Paganin phase retrieval processing^56^. Thus, being the different samples comparable in terms of size and composition, the superscript r was used to denote relative values for all mass density distribution parameters. The Roschger approach^53^ was used, for the first time to the authors’ knowledge, to study tissues different from bone.

Five parameters were extracted from the MDD^r^ profile of each sample: MDD^r^_peak_, the peak position of the histogram, which indicates the most frequently measured density (density with the highest number of pixels); MDD^r^_mean_, the mean relative mass density obtained from the integrated area under the curve; MDD^r^_fwhm_, the full width at half maxima of the distribution, describing the variation in density; MDD^r^_low_, the percentage of the area that has a density below the 5th percentile of the reference range; and the MDD^r^_high_, the percentage of the area that has a density above the 95^th^ percentile of the reference range. The threshold of P = 0.005 was arbitrarily chosen because it was considered a good compromise between maintaining good sensitivity for low and high values in the MDD^r^ and minimizing potential artifacts due to partial volume effect in the evaluation of MDD^r^_low_. This post-processing calculation of the MDD^r^ parameters was performed using the PeakFit software (Systat Software, San Jose, CA).

## Supplementary information

Supplementary Material

Movie 1

Movie 2
