# Supplementary material for: Advanced 3D Imaging of Uterine Leiomyoma’s Morphology by Propagation-based Phase-Contrast Microtomography
Source: Sci Rep. 2019 Jul 22;9:10580. doi: 10.1038/s41598-019-47048-0 (PMC6646365; doi:10.1038/s41598-019-47048-0)
Supplement: Supplementary file 1 — Supplementary Material [file 41598_2019_47048_MOESM1_ESM.pdf]

Advanced 3D Imaging of Uterine Leiomyoma's Morphology by Propagation-based Phase-Contrast Microtomography

Alessandra Giuliani[1], Stefania Greco[2], Serena Pacilè[3], Alessandro Zannotti[2], Giovanni Delli Carpini[1], Giuliana Tromba[3], Stefano Raffaele Giannubilo[1], Andrea Ciavattini[1], Pasquapina Ciarmela[2]

[1]Department of Clinical Science, Università Politecnica delle Marche, Ancona, Italy.

[2]Department of Experimental and Clinical Medicine, Università Politecnica delle Marche, Ancona, Italy.

[3]Elettra Sincrotrone Trieste S.C.p.A, Trieste, Italy.

Abstract

Supplementary material for the article submitted to Nature Scientific Reports. This material presents an example of slice thresholding by the Mixture Modeling Algorithm and movies related to microCT stack-sequences of 2D axial slices for healthy myometrium and leiomyoma tissues.

Supplementary material

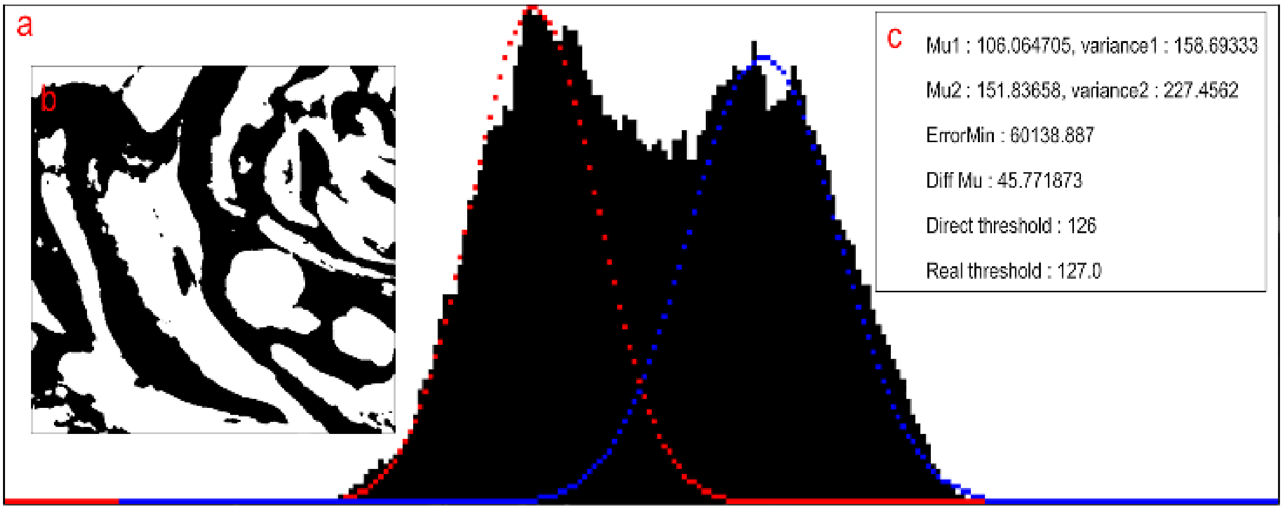

Figure 4.

Example (from P1-L biopsies) of slice thresholding by the Mixture Modeling Algorithm. The applied ImageJ plugin returns: (a) a histogram with two Gaussians (from left to right, the smooth muscle and the collagen phases), (b) the thresholded image and (c) the parameters obtained (average, standard deviation, and threshold).

Movie 1

Mv.1. Healthy myometrium. MicroCT stack-sequence of 2D axial slices (final volume of 600 × 600 × 400 μm^3) for a representative sample of P1 (P1-Ctr).

Movie 2

Mv.2. Leiomyoma. MicroCT stack-sequence of 2D axial slices (final volume of 600 × 600 × 400 μm^3) for a representative sample of P1 (P1-L).
